# *Nigella sativa* Oil Supplementation Ameliorates Symptoms and Reduces Disease Progression Biomarkers in Rats with Adjuvant-Induced Arthritis

**DOI:** 10.3390/foods15091554

**Published:** 2026-04-30

**Authors:** Anita Mihaylova, Nina Doncheva, Mariana Katsarova, Maria Vlasheva, Radoslav Tashev, Petya Gardjeva, Stela Dimitrova, Ilia Kostadinov

**Affiliations:** 1 Department of Pharmacology, Toxicology and Pharmacotherapy, Faculty of Pharmacy, Medical University of Plovdiv, 15A Vassil Aprilov Blvd, 4002 Plovdiv, Bulgaria; nina.doncheva@mu-plovdiv.bg; 2 Research Institute, Medical University of Plovdiv, 15A Vassil Aprilov Blvd, 4002 Plovdiv, Bulgaria; mariana.katsarova@mu-plovdiv.bg (M.K.); radoslav.tashev@mu-plovdiv.bg (R.T.); petya.gardjeva@mu-plovdiv.bg (P.G.); stela.dimitrova@mu-plovdiv.bg (S.D.); 3 PERIMED-2, BG16RFPR002-1.014-0007, Central District, 15A Vasil Aprilov Blvd, 4002 Plovdiv, Bulgaria; 4 Department of Bioorganic Chemistry, Faculty of Pharmacy, Medical University of Plovdiv, 15A Vassil Aprilov Blvd, 4002 Plovdiv, Bulgaria; mariya.vlasheva@mu-plovdiv.bg; 5 Department of Microbiology and Immunology “Prof. Elissay Yanev”, Faculty of Medicine, Medical University of Plovdiv, 15A Vasil Aprilov Blvd, 4002 Plovdiv, Bulgaria; 6 Laboratory of Microbiology, University Hospital St. George, 4002 Plovdiv, Bulgaria; 7 Department of Pharmacology and Clinical Pharmacology, Faculty of Medicine, Medical University of Plovdiv, 15A Vassil Aprilov Blvd, 4002 Plovdiv, Bulgaria

**Keywords:** inflammation, pain, adjuvant-induced arthritis, neuropeptide Y, IL-1β, BDNF

## Abstract

*Nigella sativa* cold-pressed oil (NSO) is rich in nutrients and biologically active compounds. This study aimed to evaluate its effects on symptoms and serum levels of inflammatory and disease activity markers in rats with Freund’s adjuvant-induced arthritis (AIA). Animals were treated orally with NSO at doses of 1 and 3 mL/kg for two weeks before arthritis induction and throughout the experiment. Hind paw edema and nociceptive thresholds were measured by plethysmometer, Hargreaves apparatus, and Randall–Selitto test, respectively. At the end of the experiment, TNF-α, IL-1β, IL-10, brain-derived neurotrophic factor (BDNF), and neuropeptide Y (NPY) serum levels were measured. NSO preventive administration significantly reduced paw edema of the affected hind paw, along with an increase in the nociceptive threshold to both thermal and mechanical stimuli. Administration of NSO resulted in a significant reduction in serum levels of IL-1β and NPY (*p* < 0.01 and *p* < 0.05, respectively), while TNF-α and IL-10 levels remained comparable to those in the untreated AIA control group. These findings indicate that NSO exerts anti-inflammatory and analgesic effects and modulates circulating IL-1β and NPY (an independent marker associated with disease activity) in experimental arthritis.

## 1. Introduction

Inflammation represents a complex and tightly regulated physiological response triggered by a wide array of stimuli, such as pathogenic microorganisms, foreign particles, physical trauma, immune system dysregulation, and exposure to chemical agents [1]. This response, while essential for host defense and tissue repair, also contributes significantly to the pathogenesis and progression of various chronic diseases, including rheumatoid arthritis (RA). It is characterized by sustained inflammation of the synovial membrane, joint swelling, autoantibody production, and gradual destruction of cartilage and bone [2]. Reactive oxygen species (ROS) and pro-inflammatory cytokines such as tumor necrosis factor-alpha (TNF-α), interleukin-6 (IL-6), and interleukin-1 beta (IL-1β) are considered key mediators in the pathogenesis of RA. These molecules contribute to disease progression by amplifying inflammatory signaling pathways, promoting the recruitment and activation of leukocytes, and inducing synovial fibroblasts to release additional cytokines and matrix-degrading enzymes. An impaired balance between inflammation-promoting and inflammation-suppressing molecules disrupts immune homeostasis, thereby facilitating the onset of autoimmunity and sustaining chronic inflammatory processes typical of RA [3].

TNF-α acts as an upstream mediator in the inflammatory cascade, initiating and amplifying immune responses that lead to joint damage. It stimulates endothelial cells and promotes the infiltration of pro-inflammatory cells, including synovial fibroblasts and macrophages, which in turn release additional pro-inflammatory cytokines such as IL-6 and IL-1β. TNF-α also regulates the differentiation of osteoclasts, T-helper (Th)1 and Th17 T cells, and antibody production [4]. IL-1β is released not only from macrophages but also from monocytes. The synthesis and activation of IL-1β play an essential role in inflammation and different pathological conditions. IL-1β stimulates nuclear factor κB (NF-κB) and mitogen-activated protein kinase (MAPK) pathways, which lead to the upregulation of other pro-inflammatory cytokines, adhesion molecules, and enzymes [5]. Elevated levels of IL-1β have been observed in patients with RA, in synovial fluid and serum. Clinical data showed that high levels of serum IL-1β were linked to RA and might be considered a potential diagnostic marker [6].

Interleukin-10 (IL-10) is a key cytokine with inflammation-suppressing and immune-regulating properties, produced by immune cells during infectious and inflammatory conditions [7]. It counteracts the progression of inflammation by downregulating NF-κB signaling and subsequent inhibition of pro-inflammatory molecules production [8]. Increased IL-10 gene expression has been detected in the peripheral blood of patients with RA, which suggests an upregulated immunomodulatory response [9].

Neuropeptide Y (NPY), a neurotransmitter of the sympathetic autonomic nervous system, may play a role in modulating cardiovascular activity, high blood pressure and obesity. NPY exhibits pleiotropic biological effects, such as modulation of brain function, emotional regulation, stress responses, appetite control, metabolic balance, and immune system function. Neuron-derived NPY can directly influence immune cells, while immune cells themselves are also capable of synthesizing and secreting NPY, allowing it to function as both a paracrine and autocrine signaling molecule within the immune system. Depending on factors such as the specific Y receptors engaged and the types of target cells involved, NPY may promote either immune activation or immune suppression [10]. Recent evidence indicates that the sympathetic nervous system plays a key function in regulating inflammation in RA. NPY may modulate the inflammatory process while acting as a mediator linking the immune and neuroendocrine networks. The serum level of NPY is considered an independent risk factor for disease activity in RA patients. This may be attributed to the activation of the promoter of this neuropeptide by TNF-α. In RA, the higher levels of NPY correlate positively with the elevated levels of TNF-α [11].

Brain-derived neurotrophic factor (BDNF) promotes neuron survival, growth, synaptic regulation, and neural plasticity. It also helps maintain and remodel neural circuits. Research shows that BDNF is important in the treatment and recovery of neurodegenerative and neurotraumatic disorders [12]. In addition, it is recognized as an important mediator of pain modulation [13]. BDNF is involved in nociceptive processing at both spinal and supraspinal levels and is associated with the pathogenesis of neuropathic and inflammatory pain. Studies showed increased expression of its receptor in chronic pain [14]. Available data indicate that serum BDNF levels are elevated in patients with RA. This factor promotes the synthesis of pro-inflammatory mediators by T lymphocytes and peripheral blood mononuclear cells [15].

*Nigella sativa* (NS), commonly known as black cumin, is a medicinal plant recognized for its broad spectrum of therapeutic properties. It is native to northern Africa, the eastern Mediterranean region, the Indian subcontinent, and Southwest Asia, and it is also cultivated in many countries across the world. NS has been widely used in traditional medicine for the management of a variety of medical conditions like hypertension, diabetes, cancer, gastrointestinal disturbances, neurological and psychiatric diseases, inflammation, etc. [16]. The plant and its derivatives possess antimicrobial, anxiolytic, anti-inflammatory, antioxidant and immunomodulatory effects, supporting its potential therapeutic applications in various pathological conditions [17]. *Nigella sativa* oil (NSO) is extracted from the plant’s seeds. It is rich in amino acids, essential fatty acids, alkaloids, minerals, sterols, and vitamins [18]. Phytochemical analyses of the oil have revealed the presence of over 35 bioactive constituents [19]. Among them are thymoquinone (TQ), dithymoquinone, thymol, and carvacrol, which are believed to contribute significantly to its biological properties [20]. The presence of both bioactive phytochemicals and vital nutrients in NS plays significant roles in normal immune function and the overall well-being, underscoring the plant’s importance as a functional nutraceutical food [16].

The biological effects of NS have been the subject of extensive research in recent years [21]. Anti-inflammatory activity represents one of the most important pharmacological characteristics of NS and its major active compound, TQ [22]. In vitro research has shown that, in human preadipocytes subjected to low-grade inflammation, treatment with NSO reduces inflammatory molecules such as IL-6 and IL-1β [23]. Furthermore, TQ inhibits the release of proinflammatory molecules, including TNF-α, IL-6, IL-1β, and nitric oxide (NO), as well as the activity of inducible nitric oxide synthase (iNOS) and cyclooxygenase-2 (COX-2) in LPS-challenged murine macrophages [24]. Accumulating evidence indicates that NS and TQ are beneficial in different types of arthritis, including RA. Reducing inflammation, suppressing oxidative stress, and regulating immune response are among the possible mechanisms through which NS can improve the overall condition in RA patients [25].

The anti-arthritic properties of NSO and TQ have been reported in several studies [25]. In patients with RA, supplementation with NSO for 8 weeks has been associated with a reduction in disease activity score and an increase in IL-10 levels [26]. Another clinical study reported that NSO reduced arthritic score, C-reactive protein, and CD8+ T cells, while increasing the percentage of CD4+ CD25+ T cells, and the CD4+/CD8+ ratio in female patients with RA [27]. Additionally, NSO has been shown to improve clinical parameters such as disease activity, joint swelling and morning stiffness in individuals with RA [28]. In animal models, NSO administration in rats with carrageenan-induced paw edema significantly decreased the levels of IL-6, IL-12 and TNF-α in paw exudates [29]. Faisal R et al. found that, in rats with pristane-induced arthritis, the effects of TQ on inflammation, synovial hyperplasia, and pannus formation were comparable to those of methotrexate [30]. In collagen-induced arthritis, TQ has been shown to alleviate joint swelling, inhibit lipid peroxidation, decrease articular elastase and myeloperoxidase activity, and enhance the activity of antioxidant enzymes such as superoxide dismutase and catalase. Joint levels of TNF-α, IL-1β, IL-6, IFN-γ, and PGE2 decreased while that of IL-10 was elevated [31].

Experimental animal models, such as adjuvant-induced arthritis (AIA) in rats, represent several specific histopathological and immunological features of human RA. Therefore, this model is widely used as a preclinical model to investigate RA pathophysiological mechanisms and to assess potential therapeutic approaches [32].

Because of its anti-inflammatory and immunomodulatory effects, NSO has attracted our interest as a potential dietary adjunct in chronic inflammatory and autoimmune conditions. However, to our knowledge, existing experimental and clinical studies have primarily focused on evaluating the effects of NSO in the presence of established arthritic symptoms or after the induction of arthritis. In this context, the present study aimed to investigate the potential preventive effects (anti-inflammatory, analgesic, and immunomodulatory) of NSO as a functional food/nutritional supplement when administered prior to arthritis induction, using an experimental model of chronic autoimmune inflammation. In addition, we explored the impact of NSO on neuroimmune markers, including NPY and circulating BDNF. Notably, no previous studies have investigated the relationship between the anti-arthritic effects of NSO and modulation of the neuroimmune axis, highlighting another important gap addressed in this work.

## 2. Materials and Methods

### 2.1. Drugs and Reagents

The following drugs and reagents were used: NSO (cold-pressed black cumin seed oil, Bioherba, Plovdiv, Bulgaria, SKU BH5431); Freund’s Adjuvant, Complete (Merck, Darmstadt, Germany, F5881-10 mL); rat IL-1β ELISA kit (Diaclone, Besancon, France, Cat. No. 670.040.096); rat IL-10 ELISA kit (Diaclone, Besancon, France, Cat. № 670.070.096); rat TNF-α ELISA kit (Diaclone, Besancon, France, Cat. № 805.000.096), rat BDNF ELISA kit (Abcam, Cambridge, UK, ab213899) and rat NPY ELISA kit (Cusabio, Wuhan, China, Cat. № CSB-E13431r).

### 2.2. High-Performance Liquid Chromatography (HPLC) Analysis of NSO

HPLC profiling of the oil was performed following the procedure previously established by our group in an earlier study [33]. The chromatographic conditions and sample preparation were identical to those previously reported. Compound detection was conducted at 254 nm for TQ and at 275 nm for thymol and carvacrol.

### 2.3. Experimental Animals and Design

In total, 24 adult male Wistar rats (200 ± 20 g body weight) were used. The animals were housed in cages (three animals per cage) and kept under standard laboratory conditions with free access to food and water. Experiments were conducted during the daytime, and the animals were habituated to the laboratory environment prior to testing

Rats were randomly assigned to four groups (*n* = 6) by an independent observer to ensure unbiased allocation: control group (C): olive oil 1 mL/kg; Complete Freund’s Adjuvant (positive) control group (CFA-C): olive oil 0.1 mL/100 g + CFA 0.1 mL; NSO-1: NSO 1 mL/kg + CFA and NSO-3: NSO 3 mL/kg BW + CFA.

Both control groups were treated with olive oil because of the oily nature of the tested substance. Experimental monoarthritis was induced by a single intraplantar injection of 0.1 mL of CFA into the right hind paw on day 15. Since the aim of the study was to evaluate the preventive effects of NSO, animals were pretreated with the oil for 14 days prior to CFA administration. NSO administration continued for three weeks following arthritis induction. At the end of the study (day 22 post-arthritis induction), blood samples were collected for subsequent immunological assay.

A reference drug group was not included, as the objective of the study was to determine whether NSO exerts a preventive effect in CFA-induced arthritis, rather than to perform comparative efficacy testing against established anti-arthritic treatments.

### 2.4. Measurement of Paw Edema

A digital water plethysmometer (Ugo Basile, Gemonio, Italy) was used to assess the CFA-induced paw edema, according to the method described by Cong et al. [34]. Hind paw volume was measured immediately before arthritis induction and on days 1, 2, 6, 9, 12, 15, 19 and 22 following the CFA injection. Paw volume was determined based on the displacement of water following immersion of the hind paw into the measuring chamber, with the displaced volume transferred to a secondary tube and recorded. The percentage of edema inhibition was subsequently calculated using the following equation:

$$\%\;inhibition\;of\;paw\;oedema=\left(\frac{PVt-PV_{0}}{PV_{0}}\right)\;\times \;100$$

where *PV*_0_ is the initial paw volume, *PVt* is the paw volume on days 1, 2, 6, 9, 12, 15, 19 and 22 following the CFA injection.

### 2.5. Nociceptive Tests

#### 2.5.1. Nociceptive Paw Pressure Test (Randall-Selitto Test; Analgesiometer)

Paw pressure threshold was assessed using an analgesiometer (Ugo Basile, Italy) according to the Randall–Selitto (RS) method. This test quantifies withdrawal responses elicited by the application of a linearly increasing mechanical force to the rat’s hind paw. Pressure was applied to the dorsum of the right hind paw and gradually increased in increments of 10 g. The endpoint was defined as paw withdrawal. A cut-off value of 250 g was imposed to prevent tissue injury. Measurements were performed on days 1, 2, 6, 9, 12, 15, 19, and 22 following arthritis induction. Baseline thresholds were recorded prior to treatment, and animals exhibiting abnormal baseline responses were excluded from the study.

#### 2.5.2. Plantar Test

Thermal nociceptive thresholds were evaluated using a Hargreaves apparatus (Ugo Basile, Italy). Animals were placed in transparent plastic chambers and allowed to acclimatize for 5 min prior to testing. A movable infrared heat source was positioned beneath the transparent floor of the chamber, delivering a focused beam with a diameter of approximately 12 mm to the plantar surface of the hind paw. The apparatus was equipped with an infrared sensor that automatically terminated heat emission upon paw withdrawal, thereby recording withdrawal latency. Measurements were performed on days 1, 2, 6, 9, 12, 15, 19, and 22 following arthritis induction. The latency to paw withdrawal was recorded. Cut off time was set at 30 s. Baseline thresholds were recorded prior to treatment, and animals exhibiting abnormal baseline responses were excluded from the study.

All anti-inflammatory and nociceptive assessments were performed by an investigator who was blinded to the experimental groups, with groups coded to ensure unbiased evaluation.

To minimize handling-induced stress, the plantar test was conducted first (with freely moving animals), followed by analgesiometer and plethysmometer assessments. This sequence was selected as analgesiometer measurements require restraint and may be influenced by handling, whereas plethysmometric evaluation of paw volume was not handling dependent.

### 2.6. Sample Collection and Immunological Assay

On day 22, the animals were euthanized, and blood samples were collected; serum was separated and stored at −70 °C until analysis. Levels of TNF-α, IL-1β, IL-10, BDNF, and NPY were measured using a solid-phase ELISA. Absorbance was recorded at 450 nm and converted into concentrations expressed as pg/mL. All ELISA assays were conducted using coded serum samples, and the investigators performing the analyses were blinded to group assignments to reduce potential bias.

### 2.7. Statistical Analysis

Statistical analyses were conducted using SPSS 17.0. Levene’s test was applied to evaluate the assumption of homogeneity of variances. When this assumption was violated (Levene’s test, *p* < 0.05), Welch’s analysis of variance (ANOVA) was employed to assess differences among groups, followed by the Games–Howell post hoc test. When homogeneity of variances was confirmed, one-way ANOVA was performed, followed by Tukey’s honestly significant difference (HSD) post hoc test. A *p*-value below 0.05 was regarded as statistically significant.

## 3. Results

### 3.1. HPLC Quantification of TQ, Thymol and Carvacrol in NSO

An HPLC method was developed for the quantification of TQ, thymol and carvacrol. The concentration of TQ in the NSO was 2%, traces of carvacrol were found, and thymol was not detected [33]. Parameters for HPLC method validation are shown in Table 1.

**Table 1:** Parameters of calibration curves, RSD, LOD and LOQ for HPLC method validation [33].

Analyte	λ (nm)	RT (min)	Regression Equations	r^2^	RSD (%)	LOD (µg/mL)	LOQ (µg/mL)	r
Thymoquinone	254	16.96	y = 3.5463 × 10^5^x	0.9974	5.92	0.640	1.935	0.9991
Thymol	275	18.31	y = 1.5218 × 10^5^x	0.9991	3.03	0.215	0.645	-
Carvacrol	275	18.56	y = 1.5941 × 10^5^x	0.9996	1.77	0.380	1.148	0.9989

**Figure 1:**
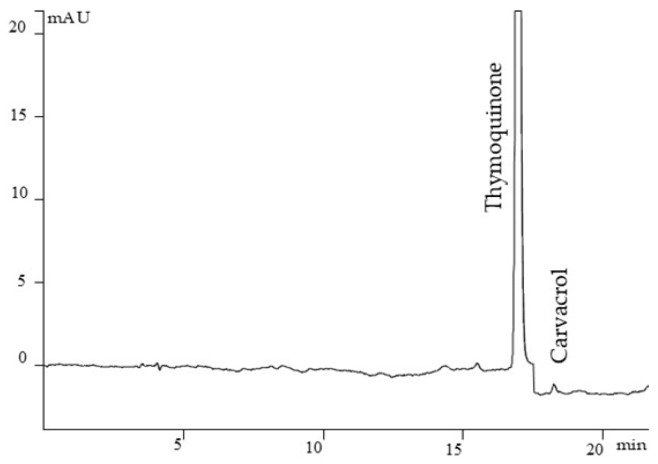
Chromatogram of NSO with detected thymoquinone and carvacrol.

Determination coefficients (r^2^) ranging from 0.9974 to 0.9996, together with RSD values between 1.77 and 5.92%, demonstrate excellent linearity of the analytical method for the quantification of TQ, thymol, and carvacrol. The high correlation coefficients (r = 0.9991 and 0.9989) reflect strong spectral congruence of the peaks associated with the target compounds. The rapid analysis time, accessibility of instrumentation, and high precision of the proposed method support its suitability for routine use. It can be effectively used for quality control of both raw materials and finished phytopharmaceutical products based on *Nigella sativa.* Figure 1 shows a chromatogram of NSO.

### 3.2. NSO Modulation of Pain and Inflammation in CFA-Induced Arthritis in Rats

#### 3.2.1. Anti-Inflammatory Test—Plethysmometer

CFA-injected control animals exhibited significantly increased paw volume compared with the control group on all test days (days 2, 6, 9, and 12: *p* < 0.01; days 1, 15, 19, and 22: *p* < 0.001), confirming the validity of the experimental model. Rats treated with the lower dose of NSO showed a gradual reduction in swelling; however, statistical significance was observed only on day 22 compared with the CFA-C group (*p* < 0.01). In contrast, treatment with the higher dose of NSO significantly reduced paw edema relative to CFA-C on all test days except day 6 (days 1 and 9: *p* < 0.05; days 2 and 12: *p* < 0.01; days 15, 19, and 22: *p* < 0.001). Furthermore, comparison between the two treatment groups revealed that NSO at 3 mL/kg produced a greater reduction in swelling than NSO at 1 mL/kg on all test days except day 6 (days 1, 2, 9, 15, and 19: *p* < 0.001; day 12: *p* < 0.01; day 22: *p* < 0.05). The results are presented in Table 2.

#### 3.2.2. Nociceptive Paw Pressure Test (Randall–Selitto Test; Analgesiometer)

Statistical analysis indicated that on the day following adjuvant injection, the paw withdrawal latency was reduced in the CFA-C group compared to the control group (*p* < 0.01). No significant differences were observed between the treated groups and the control. However, the nociceptive threshold in rats receiving both doses of NSO was higher than in the positive control group (*p* < 0.05 and *p* < 0.01, respectively). Similar results were observed on day 2: the adjuvant-treated control group exhibited a lower nociceptive threshold compared to the control (*p* < 0.05), whereas both treated groups showed higher thresholds (*p* < 0.001 and *p* < 0.01, respectively) in comparison with the CFA-C group.

This pattern persisted on days 6 and 9, with Tukey’s post hoc test showing that paw withdrawal latency in the CFA-C group was significantly shorter than in the control (*p* < 0.05), while the treated groups displayed significantly longer latencies than the positive control (*p* < 0.001 and *p* < 0.01 on day 6; *p* < 0.05 and *p* < 0.01 on day 9).

**Figure 2:**
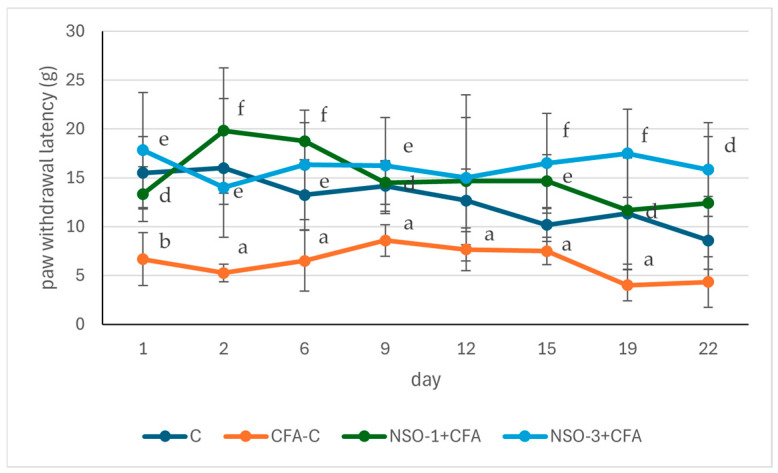
NSO-related changes in paw withdrawal latency in Randall–Selitto test. Data are expressed as mean ± SD (g). ^a^ *p* < 0.05 vs. C; ^b^ *p* < 0.01 vs. C; ^d^ *p* < 0.05 vs. CFA-C; ^e^ *p* < 0.01 vs. CFA-C; ^f^ *p* < 0.001 vs. CFA-C.

On day 12, a significantly lower nociceptive threshold was observed only in the adjuvant-treated control group compared to the control (*p* < 0.05), with no significant differences between the treated groups and the two control groups. On days 15 and 19, however, both the CFA-C group and the treated groups showed notable effects: the CFA-C group had a shorter withdrawal latency than the control (*p* < 0.05), and both NSO-treated groups exhibited significantly higher pain thresholds compared to the adjuvant control (day 15: *p* < 0.01 and *p* < 0.001, day 19: *p* < 0.05 and *p* < 0.001). On day 22, statistical significance was observed only between the NSO-3-treated group and the positive control (*p* < 0.05). These results are summarized in Figure 2.

#### 3.2.3. Plantar Test (Hargreaves’ Method)

Statistical analysis showed that on the day following adjuvant injection, the nociceptive threshold was higher in all experimental groups compared to the control group, with the strongest level of significance observed in the CFA-C and NSO 1 mL/kg groups (*p* < 0.001) and a lower level of significance in the NSO 3 mL/kg group (*p* < 0.05). No significant differences were found when comparing the treated groups with the positive control group, nor among the treated groups themselves. Similar observation was registered on day 2, when a significantly lower nociceptive threshold was recorded in the CFA-C, NSO 1 mL/kg, and NSO 3 mL/kg groups compared to the control group (*p* < 0.001, *p* < 0.01, and *p* < 0.05, respectively).

On day 6, the lower nociceptive threshold in the positive control group compared to the control group persisted (*p* < 0.05); however, no significant differences were observed between the treated groups and both control groups.

**Figure 3:**
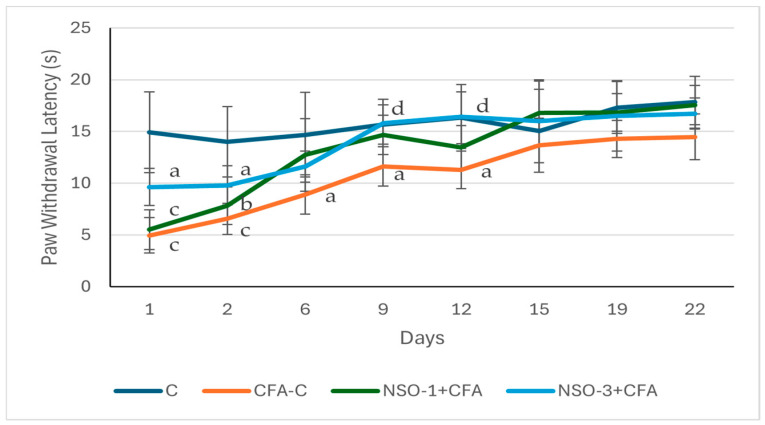
Effect of NSO on paw withdrawal latency in Plantar test. Data are expressed as mean ± SD (s). ^a^ *p* < 0.05 vs. C; ^b^ *p* < 0.01 vs. C; ^c^ *p* < 0.001 vs. C; ^d^ *p* < 0.05 vs. CFA-C.

On days 9 and 12, animals in the CFA-C group continued to show a significantly shorter latency time compared to the control group (*p* < 0.05), while rats treated with the higher NSO dose demonstrated a higher pain threshold compared to the adjuvant-treated control group (*p* < 0.05). No statistically significant difference was found between groups on days 15, 19 and 22. These results are summarized in Figure 3.

### 3.3. Impact of NSO on Serum IL-10, IL-1β, TNF-α, NPY, and BDNF Levels in CFA-Induced Arthritis

#### 3.3.1. IL-10

**Figure 4:**
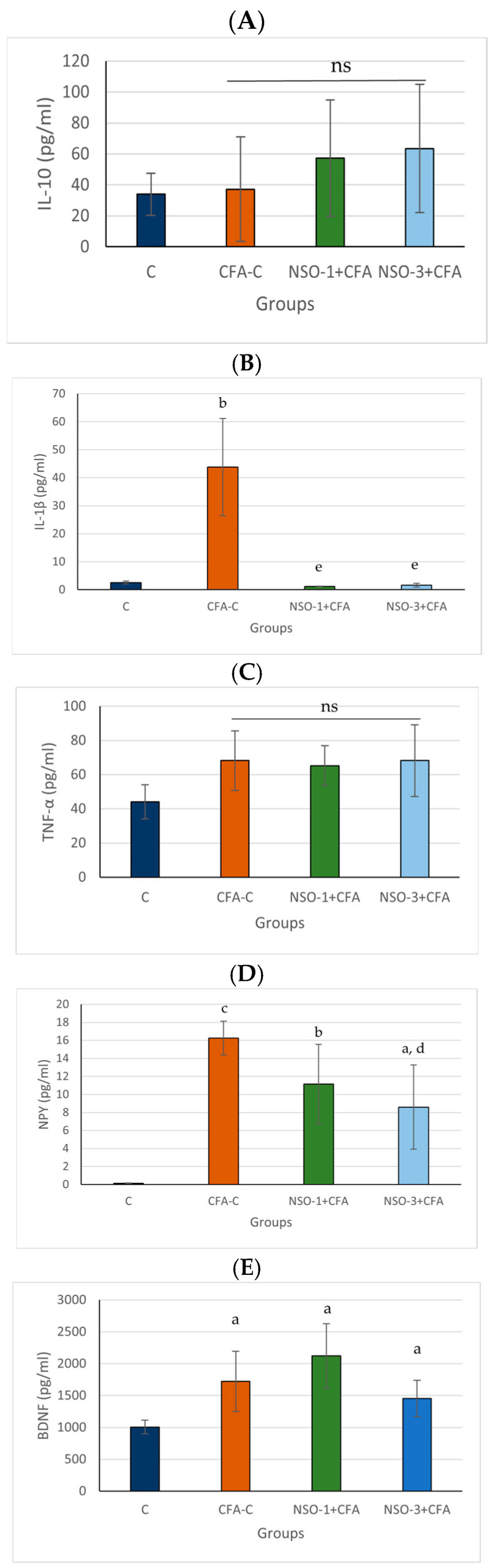
Impact of NSO on circulating IL-10, IL-1β, TNF-α, NPY, and BDNF levels in CFA-induced arthritis. (**A**). IL-10, (**B**). IL-1β, (**C**). TNF-α, (**D**). NPY, and (**E**). BDNF. Data are expressed as mean ± SD. ^a^ *p* < 0.05 vs. C; ^b^ *p* < 0.01 vs. C; ^c^ *p* < 0.001 vs. C; ^d^ *p* < 0.05 vs. CFA-C; ^e^ *p* < 0.01 vs. CFA-C; ns—not significant vs. C.

Although the positive control group and both NSO-treated groups exhibited slightly higher IL-10 levels than the vehicle group, these differences did not reach statistical significance (Figure 4A).

#### 3.3.2. IL-1β

Serum IL-1β levels in the positive control group were significantly higher than in the vehicle group (*p* < 0.01). Both experimental groups receiving NSO showed a marked decrease in serum IL-1β levels compared with the positive control group (*p* < 0.01) (Figure 4B).

#### 3.3.3. TNF-α

Serum TNF-α levels were elevated in the CFA-C group and both NSO-treated groups compared with the control group; however, these differences were not statistically significant (Figure 4C).

#### 3.3.4. NPY

Serum NPY levels in the CFA-C group were significantly increased compared with the vehicle group (*p* < 0.001). Both experimental groups showed markedly elevated NPY levels in comparison with the control group (*p* < 0.01 and *p* < 0.05, respectively). Animals treated with a higher dose of 3 mL/kg NSO showed a significant decrease in serum NPY levels compared with the positive control group (*p* < 0.05) (Figure 4D).

#### 3.3.5. BDNF

BDNF levels were significantly elevated in the CFA-C group (*p* < 0.05) as well as in both NSO-treated groups (*p* < 0.05) compared with the vehicle group (Figure 4E).

## 4. Discussion

The results of the present study demonstrated that NSO administration before arthritis induction exhibited anti-inflammatory and analgesic effects, manifested by a reduction in paw edema, as well as an increase in pain threshold in response to mechanical and thermal stimulation. To clarify the possible mechanism underlying the observed anti-inflammatory and anti-hyperalgesic effect, we examined serum levels of molecules involved in the promotion and suppression of inflammation. The results showed that NSO reduced serum IL-1β concentrations. This effect most likely plays an important role in mediating the anti-inflammatory action of the oil in settings of inflammation. Another possible mechanism is associated with a decrease in serum levels of NPY. To our knowledge, no other study in the available literature has investigated the effect of the oil on this marker.

The anti-arthritic effects of NSO have been demonstrated in animal models of AIA. Complete Freund’s adjuvant (CFA) is commonly used to induce autoimmune inflammation that closely resembles the pathogenesis and clinical manifestations of RA in humans [35]. Nasuti C et al. reported that treatment with NSO for 25 days, initiated at the onset of CFA-induced arthritis, prevented disease development and exerted beneficial effects during the acute phase, as evidenced by a significant reduction in swelling of the injected hind paw. However, NSO treatment did not influence arthritis severity during the chronic phase or serum IL-6 levels [36]. Our results are in agreement with these observations. In the current study, NSO supplementation for two weeks prior to arthritis induction prevented its development, as demonstrated by reduced paw edema of the injected hind paw. Notably, our experimental design differs from that of previous studies by employing a preventive rather than therapeutic approach, which may further explain the observed effects.

In contrast, another study reported that NSO decreased paw edema in rats with carrageenan-induced inflammation but had no significant effect on animals with AIA [37]. This discrepancy with our findings is likely attributable to differences in treatment duration, as NSO was administered only for 7 days (either before or after arthritis induction) in that study. These findings suggest that the duration and timing of NSO administration may be critical determinants of its anti-arthritic efficacy.

The antiarthritic effect of NSO is probably related to TQ. The latter is considered the main biologically active compound found in NS seeds. Our previous research showed high TQ concentration in the oil. In rats with CFA-induced arthritis, TQ in a dose of 10 mg/kg reduced arthritic score, pannus formation, cellular infiltration, and decreased C-reactive protein as well as mRNA expression of Toll-like receptors (TLR2 and TLR4), the transcription factor NF-κB, and cytokines IL-1 and TNF-α [38]. In human osteoarthritis chondrocytes stimulated with IL-1β, TQ inhibited the production of matrix metallopeptidases, inflammatory molecules and enzymes, responsible for their synthesis (PGE2, NO, COX-2 and iNOS) through the inhibition of NFκB and MAPK [39]. In RA synovial fibroblasts, TQ inhibited TNF-α-induced production of inflammatory molecules (IL-6 and IL-8) and adhesion molecules by suppressing apoptosis-regulated signaling kinase 1 [40]. The molecular mechanisms through which TQ contributes to the treatment of autoimmune diseases are summarized by Ali MY et al. [41].

The present work also sought to evaluate the analgesic properties of NSO in the inflammation setting. As expected, the pain threshold to both thermal and mechanical stimuli was notably decreased in animals from the CFA-C group. NSO demonstrated a more pronounced antinociceptive effect in response to mechanical stimulation, as assessed by the Randall–Selitto test, compared with thermal stimulation in the plantar test. Within the Randall–Selitto paradigm, the analgesic effect of NSO was evident from the early days following inflammation induction at both tested doses. Moreover, this effect persisted throughout the experiment, with statistical significance maintained until the final assessment, at which point it was detected only at the higher dose. In contrast, in the plantar test, the onset of the analgesic effect was delayed (day 9) and was registered only at the 3 mL/kg dose. Our findings are broadly consistent with previous studies, while also extending them. An earlier study reported an analgesic effect of NS seeds’ crude suspension against thermal stimuli in mice with carrageenan-induced edema; however, this effect was demonstrated only after acute administration [42]. In contrast, our results show that repeated preventive NSO administration produces a sustained analgesic effect under chronic inflammatory conditions. Similarly, in a rat model of chronic constriction injury of the sciatic nerve, chronic—but not acute—NSO treatment reduced both heat hyperalgesia and mechanical allodynia, supporting the importance of prolonged administration for achieving analgesic efficacy [43]. Our findings are in agreement with this observation, particularly regarding the sustained attenuation of mechanical hyperalgesia. Furthermore, Pop et al. demonstrated that in AIA, both preventive and therapeutic NSO administration (7 days before or after arthritis induction) attenuated mechanical hyperalgesia, whereas effects on thermal hyperalgesia were observed only following preventive administration and only transiently [37]. These results closely align with our data, which also indicate a more robust and sustained effect on mechanical nociception compared with thermal sensitivity.

Pain models based on thermal stimuli primarily assess centrally mediated mechanisms of analgesic action [44]. In contrast, the Randall–Selitto test is considered more appropriate for the evaluation of peripheral analgesic activity, as it measures the response to mechanical pressure applied to inflamed tissue [45]. Based on these methodological differences, it may be speculated that the analgesic effect observed in the present study is more likely related to the inhibition of peripheral inflammatory processes rather than to direct modulation of central nervous system structures. Specifically, the reduction in nociceptive sensitivity may result from attenuation of inflammatory mediators and decreased activation of peripheral nociceptors in the affected tissues.

Multiple inflammatory mediators and cells are involved in the pathogenesis of RA. Among them, TNF-α and IL-1β are some of the most well-studied and play a key role in disease progression [46]. Some of the most effective contemporary therapies for this condition are targeted against these two cytokines [47]. Anti-TNF-α strategies have a leading role, but data on the efficacy of anti-IL-1β therapies are also promising [48]. Accordingly, we investigated the effect of NSO on circulating TNF-α and IL-1β, as well as IL-10, which has a counter-regulatory role and limits the development of the inflammatory response. Despite the literature data reporting anti-arthritic activity of NS in CFA-induced models, these studies do not include monitoring the effect on the aforementioned cytokines. Nasuti and colleagues have investigated the effect of NSO on IL-6 levels in AIA models and reported that treatment did not significantly influence the inflammatory cytokine IL-6 [36]. This cytokine also plays a key role in the pathogenesis of RA, but due to available data, we did not include it in the cytokine panel in the present study.

The evidence supporting the influence of NSO on TNF-α, IL-1β, and IL-10 is provided by numerous experimental studies demonstrating its immunomodulatory properties [49]. However, data regarding its effect on TNF-α production remain inconsistent. NSO supplementation decreases serum TNF-α levels in various preclinical and clinical studies. For instance, Razmpoosh E et al. found that NSO inhibits TNF-α mRNA expression in peripheral blood mononuclear cells and reduces TNF-α serum levels in obese and overweight women [50], while reductions have also been observed in experimental models of colitis [51]. In contrast, other studies suggest that NS may exert immunostimulatory effects, including increased cytokine production such as TNF-α [52].

In the present study, NSO administration was associated with a tendency toward decreased TNF-α and increased IL-10 levels; however, these changes did not reach statistical significance. This finding may be explained by several factors. The effect of NSO on TNF-α appears to be highly context-dependent, varying according to the type and localization of the inflammatory stimulus. In addition, TNF-α is an early-phase cytokine with transient expression, and its levels are strongly influenced by the timing of measurement. Importantly, the relatively small sample size in the current study may have limited the statistical power, thereby contributing to the lack of statistical significance despite observable trends.

However, we found that pretreatment with black cumin oil significantly reduced IL-1β levels in an experimental model of arthritis. It is well known that this cytokine plays an important role in the pathogenesis of RA, and its serum levels are elevated in patients with this disease [53]. NS and especially its active compound TQ have consistently demonstrated anti-inflammatory action through reduction in IL-1β in both experimental and some human studies. Experimental studies demonstrate that NS or TQ administration significantly reduces IL-1β levels, along with other pro-inflammatory mediators such as TNF-α and IL-6, while promoting anti-inflammatory cytokines like IL-10. That effect is most likely mediated by suppression of key NF-κB signaling, reduction in oxidative stress, and modulation of macrophage activation, all of which contribute to the attenuation of joint inflammation and tissue damage [54,55]. Xing et al. demonstrated that TQ decreased the expression of inflammatory factors such as TNF-α, IL-1β, and IL-6 in mice with paclitaxel-induced neuropathy [56]. A clinical crossover trial in overweight patients showed that daily NSO supplementation for 2 months significantly reduced blood IL-1β levels and IL-1β gene expression [50], supporting its potential as a complementary therapeutic strategy in inflammatory conditions and obesity.

In our study, prophylactic administration of NSO significantly decreased serum NPY levels in rats with CFA-induced arthritis compared with the CFA group. This finding indicates a modulatory effect of NSO on circulating NPY under inflammatory conditions. NPY is known to participate in neuroimmune regulation and pain signaling, and elevated synovial and circulating levels in individuals with RA have been associated with greater pain intensity, higher disease activity, and elevated TNF-α expression [11,57]. Immune cells express multiple NPY receptor subtypes, indicating their ability to respond to NPY signaling [10,58]. Furthermore, González-Chávez et al. reported a positive association between serum NPY levels and disease severity in a study using rapamycin to lower arthritis incidence. NPY knockdown has been shown to decrease the expression of pro-inflammatory cytokines, including TNF-α, IL-1β, and IL-6, providing additional evidence for functional communication between sympathetic neuropeptide signaling and immune cells [59]. Accordingly, the reduction in NPY observed in the present study may reflect attenuation of inflammation-associated neuropeptide activity.

Overall, these results suggest that NSO may influence NPY-related neuroimmune signaling in CFA-induced arthritis. Although studies directly examining NPY modulation by NSO in arthritis are missing, evidence from other inflammatory disease models suggests that NSO can regulate levels of inflammatory mediators and neuroinflammation, which may influence neuro-immunomodulatory peptides such as NPY [60]. However, given the limited mechanistic scope of the present study, additional studies are needed to elucidate the pathways underlying this effect.

In our study, preventive NSO administration resulted in a non-significant reduction in serum BDNF levels in rats with experimental AIA. Given that peripheral BDNF has been reported to be associated with disease activity and inflammation in RA [61], this trend may reflect a possible effect of NSO on neuroimmune-related pathways; however, this cannot be conclusively determined from the present data. The anti-inflammatory and antioxidant properties of NSO may contribute to the modulation of cytokine production and immune cell activity, which in turn could regulate BDNF expression [62]. Previous studies have shown that suppression of pro-inflammatory mediators is associated with decreased BDNF levels in inflammatory conditions [63]. In the present study, the observed decrease did not reach statistical significance, likely due to limited sample size and reduced statistical power. Further studies with larger cohorts and targeted mechanistic designs are therefore warranted.

Several limitations of our study should be noted. First, only two doses of NSO were investigated, which limits the ability to establish a comprehensive dose–response relationship. Future studies should include a broader range of doses to better characterize the pharmacological profile of the oil and identify the optimal effective dose. Second, the relatively small sample size limits the generalizability of the findings. Third, a control group treated with a reference anti-inflammatory drug was not included. The study was designed primarily to evaluate the intrinsic biological activity of NSO in the adjuvant-induced arthritis model rather than to compare its efficacy with established therapies. However, comparative studies with standard anti-inflammatory or disease-modifying agents would be valuable for better understanding the potential therapeutic relevance of NSO and its possible role as an adjunct to conventional treatment. Finally, histopathological evaluation of paw and joint tissues was not performed, which limits the ability to directly correlate the observed functional (paw edema and nociceptive thresholds) and immunological changes with structural alterations in the affected tissues. Additional studies at the tissue or molecular level (e.g., receptor expression and local mediator production) would be necessary to further evaluate the neuroimmunomodulatory activity of NSO in the setting of autoimmune inflammation. Despite these limitations, the present results provide important evidence supporting the anti-inflammatory and analgesic potential of NSO.

## 5. Conclusions

The present study demonstrates that pretreatment with NSO may alleviate pain and inflammation in an experimental model of arthritis. The observed anti-inflammatory and anti-hyperalgesic effects are likely related to the immunomodulatory properties of biologically active compounds present in the oil. Our results showed that NSO reduced serum levels of IL-1β without significantly affecting those of TNF-α and IL-10. Moreover, to our knowledge, this study demonstrates for the first time that NSO decreases serum concentrations of NPY, which has been identified as an independent risk factor for disease progression in patients with RA. This finding may be associated with both the anti-inflammatory and analgesic properties of the oil and opens a new avenue for future research aimed at elucidating the mechanisms underlying the biological activity of NSO.

## Figures and Tables

**Table 2:** Effect of NSO on paw volume in CFA-induced arthritis in rats.

	C	CFA-C	NSO-1	NSO-3
Day 1	1.67 ± 1.1	82.42 ± 17.81 ^c^	102.95 ± 14.45 ^c^	47.81 ± 5.7 ^c,d,i^
Day 2	1.67 ± 1.1	69.23 ± 20.47 ^b^	84.48 ± 14.84 ^c^	40.43 ± 5.98 ^c,e,i^
Day 6	1.67 ± 1.1	54.16 ± 20.51 ^b^	48.58 ± 16.07 ^b^	24.73 ± 7.89 ^b^
Day 9	1.67 ± 1.1	55.90 ± 19.23 ^b^	50.83 ± 5.85 ^c^	19.65 ± 8.23 ^a,d,i^
Day 12	1.67 ± 1.1	59.52 ± 14.28 ^b^	51.75 ± 12.75 ^b^	15.70 ± 7.38 ^a,e,h^
Day 15	1.67 ± 1.1	59.45 ± 10.99 ^c^	47.92 ± 10.53 ^c^	9.96 ± 4.36 ^b,f,i^
Day 19	1.67 ± 1.1	59.45 ± 10.99 ^c^	47.91 ± 10.52 ^c^	7.91 ± 3.53 ^a,f,i^
Day 22	1.67 ± 1.1	54.13 ± 9.19 ^c^	24.72 ± 10.58 ^a,e^	4.87 ± 3.63 ^f,g^

Data are expressed as mean ± SD (% inhibition of paw edema). ^a^ *p* < 0.05 vs. C; ^b^ *p* < 0.01 vs. C; ^c^ *p* < 0.001 vs. C; ^d^ *p* < 0.05 vs. CFA-C; ^e^ *p* < 0.01 vs. CFA-C; ^f^ *p* < 0.01 vs. CFA-C; ^g^ *p* < 0.05 vs. NSO-1; ^h^ *p* < 0.01 vs. NSO-1; ^i^ *p* < 0.001 vs. NSO-1.

## Data Availability

The original contributions presented in this study are included in the article. Further inquiries can be directed to the corresponding authors.

## Abbreviations

The following abbreviations are used in this manuscript:

AIA	Adjuvant-induced arthritis
BDNF	Brain-derived neurotrophic factor
COX	Cyclooxygenase
ELISA	Enzyme-linked immunosorbent assay
IL-1β	Interleukin-1 beta
IL-6	Iinterleukin-6
IL-10	Interleukin-10
iNOS	Inducible nitric oxide synthase
MAPK	Mitogen-activated protein kinase
NS	*Nigella sativa*
NSO	*Nigella sativa* oil
NO	Nitric oxide
NF-κB	Nuclear factor kappa-light-chain-enhancer of activated B-cells
NPY	Neuropeptide Y
RA	Rheumatoid arthritis
ROS	Reactive oxygen species
TLR	Toll-like receptors
TNF-α	Tumor necrosis factor-alpha
Th	T-helper
TQ	Thymoquinone
